# A review of the methods used to define glucocorticoid exposure and risk attribution when investigating the risk of fracture in a rheumatoid arthritis population

**DOI:** 10.1016/j.bone.2016.06.001

**Published:** 2016-09

**Authors:** D.E. Robinson, E.M. Dennison, C. Cooper, T.P. van Staa, W.G. Dixon

**Affiliations:** a Arthritis Research UK Centre for Epidemiology, Centre for Musculoskeletal Research, Institute for Inflammation and Repair, Manchester Academic Health Science Centre, University of Manchester, Manchester, M13 9PT, UK; b MRC Lifecourse Epidemiology Unit, University of Southampton, Southampton General Hospital, Tremona Road, Southampton SO16 6YD, UK; c Victoria University, Wellington, New Zealand; d NIHR Musculoskeletal Biomedical Research Unit, Nuffield Department of Orthopaedics, Rheumatology and Musculoskeletal Sciences, University of Oxford, Oxford OX3 5UG, UK; e NIHR Nutrition Biomedical Research Centre, University of Southampton and University Hospital Southampton NHS Foundation Trust, Southampton General Hospital, Southampton SO16 6YD, UK; f Health eResearch Centre, Farr Institute for Health Informatics Research, University of Manchester, Vaughan House, Portsmouth Road, M13 9PL, UK; g Utrecht University, Faculty of Science, Division of Pharmacoepidemiology and Clinical Pharmacology, Utrecht, The Netherlands; h NIHR Manchester Musculoskeletal Biomedical Research Unit, Central Manchester University Hospitals NHS Foundation Trust, Manchester Academic Health Science Centre, Nowgen Building, 29 Grafton Street, Manchester, M13 9WU, UK; i Salford Royal NHS Foundation Trust, Stott Ln, Salford, M6 8HD, UK

**Keywords:** Glucocorticoids, Rheumatoid arthritis, Fracture, Rheumatology, Epidemiology

## Abstract

**Background:**

Glucocorticoid therapy is used widely in patients with rheumatoid arthritis (RA) with good efficacy but concerns about safety including fractures. Estimates of fracture risk for any given patient are complicated by the dynamic pattern of glucocorticoid use, where patients vary in their dose, duration and timing of glucocorticoid use.

**Objective:**

To investigate which methods are currently used to attribute fractures to glucocorticoid exposure and investigate whether such methods can consider individual treatment patterns.

**Results:**

Thirty-eight studies used five common definitions of risk attribution to glucocorticoid exposure: “current use”, “ever use”, “daily dose”, “cumulative dose” and “time variant”. One study attempted to combine multiple definitions where “cumulative dose” was nested within “daily dose”, covering the effects of dose and duration but not timing. The majority of results demonstrated an equivocal or increased risk of fracture with increased exposure, although there was wide variation, with odds ratios, hazard ratios and relative risks ranging from 0.16 to 8.16. Within definitions there was also variability in the results with the smallest range for “time variant”, 1.07 to 2.8, and the largest for “cumulative dose”, ranging from risk estimates of 0.88 to 8.12.

**Conclusion:**

Many studies have looked into the effect of glucocorticoids on fracture risk in patients with RA. Despite this, there is no clear consensus about the magnitude of risk. This is a consequence of the varied analysis models and their different assumptions. Moreover, no current analysis method allows consideration of dose, duration and timing of glucocorticoid therapy, preventing a clear understanding of fracture risk for patients and their individual treatment patterns.

## Highlights

• A literature review on which methods are used to define risk attribution of glucocorticoid use to fractures was undertaken.
• Five common methods of defining risk attribution of glucocorticoids were found.
• No currently used method considers dose, duration and timing.
• Dose, duration and timing are thought to affect the risk of fracture due to the dynamic patterns of glucocorticoid therapy.

## 1 Introduction

Rheumatoid arthritis (RA) is an autoimmune disease which affects between 0.5 and 1% of the population [1]. Glucocorticoids were identified as a treatment for RA over 60 years ago [2] and approximately 2/3 of patients have ever used glucocorticoids [3]. They have been found to reduce joint tenderness and pain [4], and to reduce the rate of disease progression when used in addition to standard therapies [5]. Whilst reducing disease progression, there are adverse effects associated with the use of glucocorticoids, including bone fracture, infection, cataracts, and diabetes [6]. Glucocorticoid use tends to be dynamic, with patients switching between periods of use and non-use, and with varying doses through time in response to their disease severity. Thus most patients have a personalised treatment plan.

Glucocorticoids primarily affect bone health and fracture risk by acting on functions critical in the regeneration and healing cycles [7]. Glucocorticoids have been shown to affect the function of both osteoclasts and osteoblasts, resulting in disruption to bone repair. This impact on bone remodelling weakens the bone making it more brittle and at higher risk of fracture [8]. The brittleness of the bones also causes a reduction in bone mineral density (BMD) for those on glucocorticoids and hence an increase in the risk of fracture has been found at levels of BMD where the patient does not have osteoporosis [9] suggesting they affect fracture risk above and beyond the usual effect of decreasing BMD. Despite the acceptance that glucocorticoid therapy increases the risk of fracture, estimates about the size of the effect vary widely. This may be because a wide range of definitions has been used to attribute fractures to glucocorticoid exposure.

It is likely that the impact of glucocorticoids on fracture risk relates to the dose administered, the duration of exposure, the latency between administration and effect on bone regeneration, and post-exposure recovery [10]. This review will therefore investigate the range of different definitions used to attribute fractures to glucocorticoid exposure in patients with RA, and investigate the impact of these different definitions on the results. The assumptions of each definition of glucocorticoid exposure will be assessed for suitability with regards to the dynamic patterns of glucocorticoid exposure experience by patients with RA by reviewing their consideration of dose, duration and timing.

## 2 Methods

A literature search was carried out in Ovid using the databases Medline and Embase. For both databases the years covered by the search were from the conception of the database until the end of October 2014.

The search criteria used for inclusion of papers included terms for glucocorticoids, fractures, and RA, (see Appendix) and were the same for both databases. Searches were initially limited to English language, humans, adults with further limitations made to the publication type. In Medline, publication types removed included: case reports, Phase I or II clinical trials, reviews, meta analyses, duplicate publications, retracted publications and any other non-research article publications. In Embase, publication types removed included books, book series, conference papers, editorials, notes, reviews and short surveys. Following exclusions, abstracts were screened to ensure the topic of the paper was suitable and if no abstract was available within Ovid, attempts were made to find the paper online.

## 3 Results

**Fig. 1:**
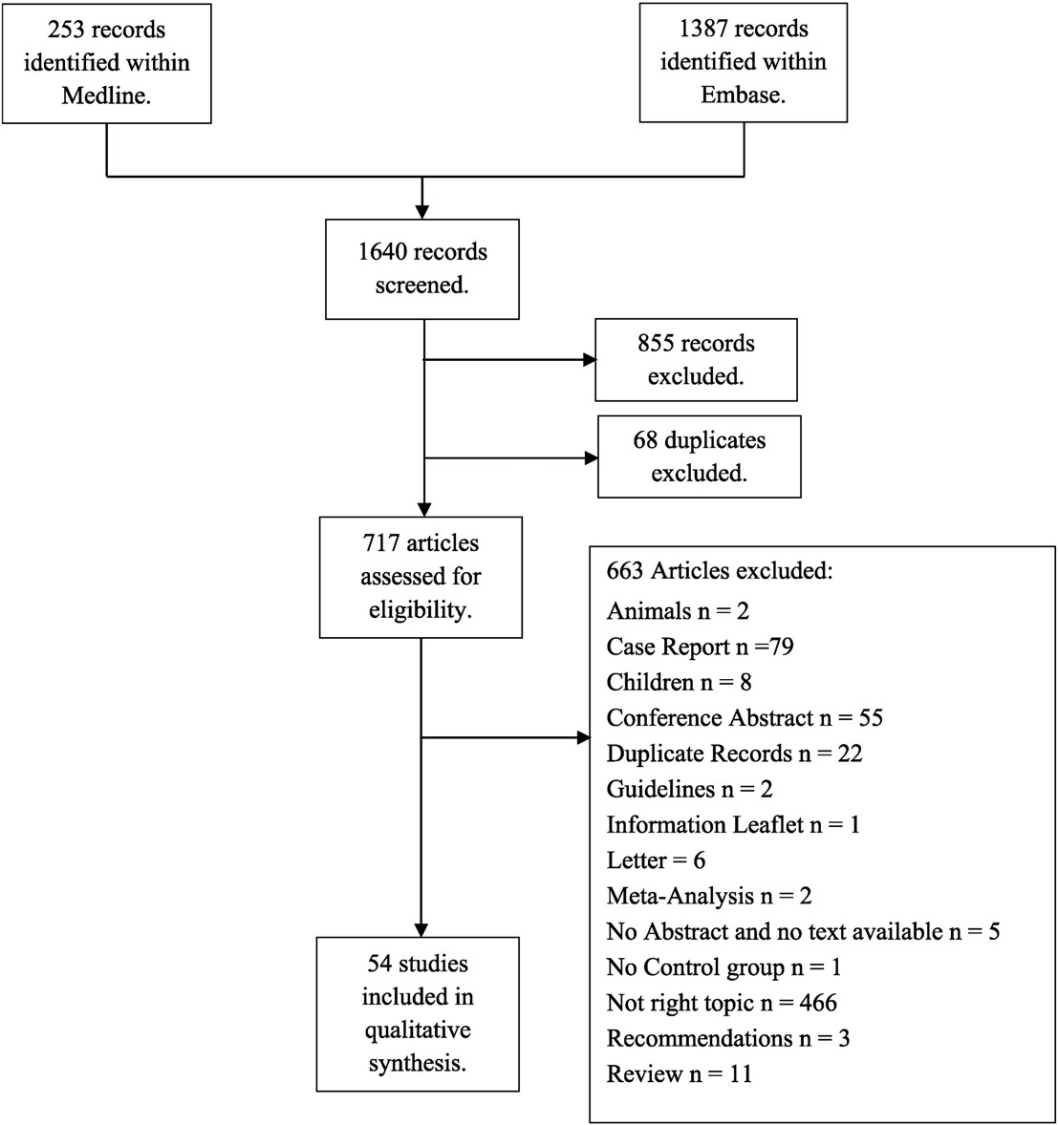
Flow diagram.

Fig. 1 describes the number of papers found at each stage of the literature search with the reasons papers were discarded.

During a qualitative synthesis stage, a further 16 papers were excluded either because there was no analysis of effect of glucocorticoids on fracture reported (5 papers), only 1 fracture occurring in either the glucocorticoid exposed or unexposed cohorts (2 papers), no association between glucocorticoids and fracture was reported (5 papers), RA was not considered in the paper (2 papers) or if the English full text version was not available (2 papers).

**Fig. 2:**
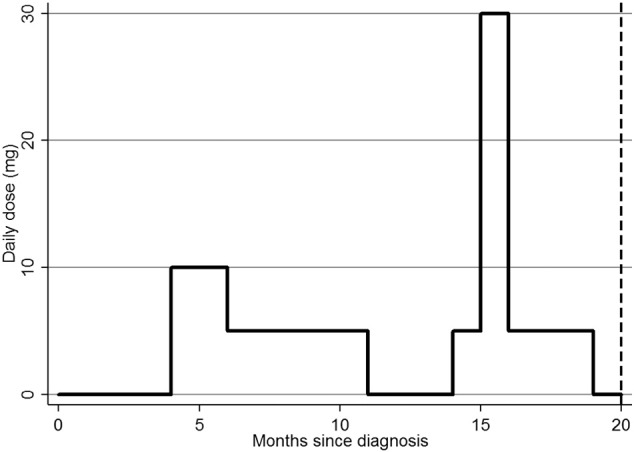
A hypothetical patient's exposure to glucocorticoids. Footnote: at 20 months: current use = No, ever use = Yes, daily dose = 0 mg, cumulative dose = 2.85 g, time variant = 12 months on glucocorticoids.

Thirty eight papers were selected for review (Table 1). The definitions for attributing fractures to glucocorticoid exposure were as follows: “current use” (n = 19), “ever use” (n = 15), “daily dose” (n = 13), “cumulative dose” (n = 8), “multi-variable” (n = 2), “time variant” (n = 8) and other definitions of glucocorticoid exposure (n = 3). Multiple definitions were reported in 17 papers. These models are described further in Fig. 2 using a hypothetical dynamic exposure pattern for an individual patient.

### 3.1 Descriptions with binary response

Two definitions of glucocorticoid exposure were binary variables. Firstly the “current use” definition, which meant the participant was on glucocorticoids at the time of fracture and secondly the “ever use” definition, which attributed an incident fracture to glucocorticoid use if a participant had ever taken glucocorticoids during the study period. In the exposure pattern for a hypothetical patient shown in Fig. 2, “current use” at the time of fracture would be 0 (i.e. the patient is off drug at the time of fracture and the fracture is thus not attributed to their glucocorticoid exposure) and “ever exposed” would be 1 (i.e. they HAVE ever been exposed to glucocorticoid therapy and the fracture would be attributed to their historical exposure.)

Of the 19 papers that reported results for “current use” of glucocorticoids, nine [15], [16], [17], [18], [19], [20], [25], [26], [28], [29] found a statistically significant increase in the risk of fracture, five [11], [14], [21], [22], [23] found no significant change in the risk of fracture, one [24] found a decreased risk of fracture, one found an increased risk in women but not men and the remaining three [12], [13], [27] found an increased risk at some fracture sites but not others. Those who found an increased risk of fracture had an odds ratio, relative risk or hazard ratio between 1.33 [13] and 4.15 [15] for current users compared to non-users whereas the paper that reported a decreased risk had an odds ratio of 0.17 for current users compared to the general population [24].

Within the 15 papers reporting “ever use” of glucocorticoids, six [20], [21], [30], [33], [37], [41] found an increased risk of fracture, five [31], [32], [36], [38], [40] found no statistically significant change in the risk of fracture, two [34], [39] found an increased risk at some fracture sites but not others, one [19] found an increased risk for certain age groups and one [35] reported an increased risk in women but not men. Within the six papers that reported an increased risk, only two reported odds ratios, relative risk or hazard ratios with values of 1.69 [35] and 8.16 [41].

**Fig. 3:**
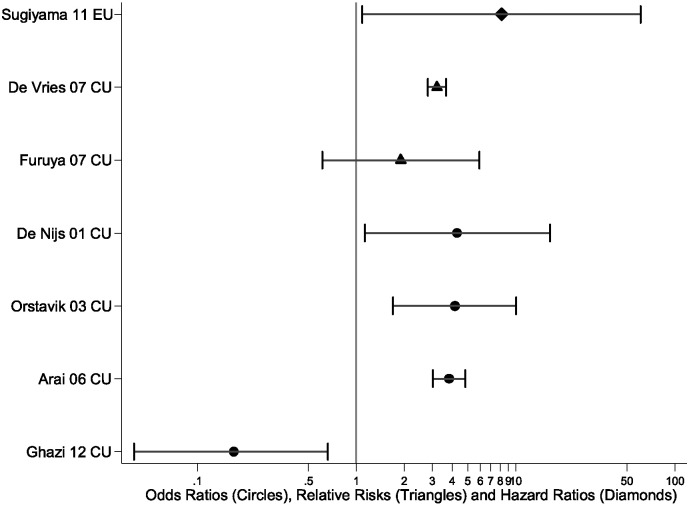
The odds ratios (circles), relative risks (triangles) and hazard ratios (diamonds) with 95% confidence intervals showing the information given from the two binary methods, current use and ever use using results defining vertebral fractures.

Fig. 3 illustrates the range of results obtained for both the “current use” and “ever use” methods of attributing fractures to glucocorticoid exposure, limited to vertebral fractures. A single fracture type was selected to reduce other causes of heterogeneity, enabling a comparison of the impact of analysis methodology on the results. Vertebral fractures were chosen since the majority of papers (29/39 studies (74%)) reported on either clinical or radiographic vertebral fractures.

Fig. 3 demonstrates that the increased risk of vertebral fracture was not consistent within the “current use” definition, with one “current use” paper having a relative risk around 2, with one “current use” paper having a relative risk around 3, and three “current use” papers having an odds ratio about 4. Fig. 3 demonstrates that the increased risk of vertebral fracture was not consistent within the “current use” definition, with one “current use” paper having a relative risk around 2, with one “current use” paper having a relative risk around 3 Three “current use” papers had an odds ratio about 4 whilst one had an odds ratio of around 0.2. A meta-analysis of these odds ratios for “current use” would generate approximately a fourfold increased risk of vertebral fracture. Furthermore, Fig. 3 illustrates through their absence that 12/15 publications considering “ever use” reported P-values only and no point estimate, and are hence missing.

### 3.2 Descriptions of dose

Two methods were used to attribute fractures to definitions of glucocorticoid exposure which demonstrated the effect of dose. Firstly the “daily dose” definition which described the dose the participant was on at the time of fracture and secondly the “cumulative dose” definition which described the total dose taken during the patients follow-up. In the hypothetical exposure pattern described in Fig. 2, the “daily dose” would be 0 mg and the “cumulative dose” 2.85 g. These two dose-specific definitions were also combined in two papers describing the effect of dose and duration in a multi-variable model.

Of the 13 papers using “daily dose” of glucocorticoid, nine [21], [23], [25], [26], [27], [37], [38], [41], [42] papers found an increased risk of fracture with increasing dose, one [40] found no statistically significant change in risk, one [22] found an increased risk for women but not men and two [24], [39] did not report statistics regarding “daily dose” although they mentioned recording “daily dose”. The odds ratios for those who found an increased risk ranged from 1.03 [22] to 2.03 [41] when “daily dose” was considered as a continuous variable (per mg per day) and 1.60 [26] (for < 7.5 mg/day compared to past use) to 4.5 [38] (for 5–10 mg/day compared to never treated patients) when “daily dose” was considered categorically.

Of the eight papers reporting “cumulative dose”, four [26], [28], [43], [44] reported an increased risk of fracture with increasing cumulative exposure, two [25], [45] reported no change to the risk of fracture and two [41], [42] reported a decreased risk of fracture. The odds ratios for those who found an increased risk ranged from 1.03 [43] to 4.31 [26] per gram increase as a continuous variable whilst those who found a decreased risk ranged from 0.88 [41] to 0.92 [42] per gram increase. When considered as a categorical variable the results ranged from 1.38 [29] if < 1 g had been consumed compared to past glucocorticoid use to 8.12 [29] when between 1 and 5 g had been consumed compared to past glucocorticoid use.

**Fig. 4:**
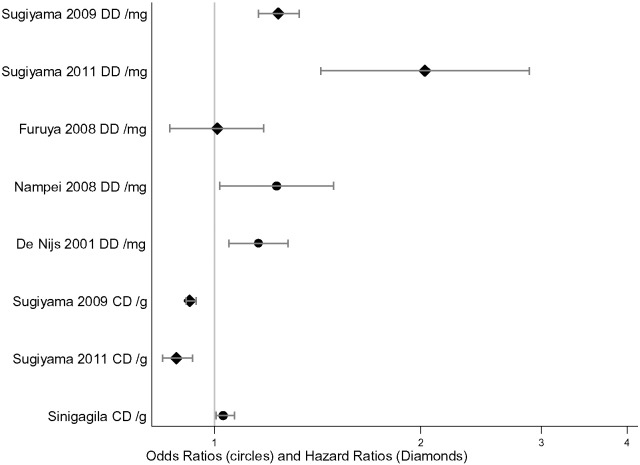
The odds ratios (circles), and hazard ratios (diamonds) with 95% confidence intervals showing the information given from the daily dose and cumulative dose methods of defining glucocorticoid exposure using results defining vertebral fractures.

A plot of the differences within estimates of fracture risk from “daily dose” and “cumulative dose” are demonstrated in Fig. 4.

Two [26], [46] papers nested “cumulative dose” within “daily dose” to provide a multi-variable model. This method showed a statistically significant increase risk of fracture for most combinations of “daily dose” and “cumulative dose” suggesting that there is a combination of effect from both “daily dose” and “cumulative dose”.

### 3.3 Descriptions of duration

One commonly used definition described duration of glucocorticoid therapy, the “time variant” definition which described the change in risk of fracture by the length of time spent on glucocorticoids. In the hypothetical exposure pattern described in Fig. 2, the duration spent on glucocorticoids was 12 month. Eight papers reported a time variant model of which four [20], [27], [44], [47] reported an increased risk of fracture with increasing exposure, three [21], [40], [45] reported no significant change and one [29] found an increased risk for those who had taken glucocorticoids for greater than 5 years compared to non- users. The odds ratios for those who found an increased risk ranged significantly from 1.07/year [47] to 2.8/month [44].

### 3.4 Other methods for attributing fractures to glucocorticoid exposure

Of the three papers who reported other methods of defining glucocorticoid exposure, one [9] investigated the effect of the number of prescriptions in the past six months and two [41], [42] investigated multiple methods of glucocorticoid exposure including pulse therapy, initial daily dose and number of dose increases.

No meta-analysis was attempted due to heterogeneity in the studies, such as the different fracture types, different comparator populations, study designs, different confounders, and small study numbers once stratified by risk attribution model.

## 4 Discussion

Five common methods were identified within this literature review that defined risk attribution of fracture risk to oral glucocorticoids in patients with RA: “current use”, “ever use”, “daily dose”, “cumulative dose” and “time-variant”. A multi-variable model was also identified where “cumulative dose” was nested within “daily dose”. Whilst the majority of papers showed an increased risk of fracture, regardless of the method defining risk attribution, the magnitude of this risk varied greatly. For example, estimates of increased risk ranged from 1.03 per gram increase in cumulative dose [43] to 8.16 [41] in patients ever exposed compared to never exposed. Conversely, the lowest estimate of risk was 0.17 [24] for patients currently using glucocorticoids compared to those not currently using glucocorticoids and suggested a protective effect. However, this contradicted most other findings. Even within analytic models, there was marked variation in the results.

The appropriateness of the analysis model is important to consider for drugs that are taken dynamically through time such as glucocorticoids (see Fig. 2).

The “current use” definition examines the association between the fracture and whether the patient was exposed to glucocorticoids on the day of the fracture, hence important assumptions for this model are that any prior glucocorticoid exposure does not affect the risk of fracture, and the dose of glucocorticoids on the day of fracture is not important. The wide range of results for “current use”, from 1.33 [13] to 4.15 [15], may reflect variability in patterns of prior use between studies. Indeed, the mean length of follow up ranged from 1 year to 12.6 years for papers reporting “current use”, allowing very different prior exposure patterns. The “ever use” definition, conversely, assumes that all historical therapy affects the risk of fracture, but this is regardless of how recently the therapy was taken. The range of results was harder to compare for this model since only P-values were reported in most cases.

The “daily dose” definition assumes that the strength of the dose on the day of fracture has the largest effect on fracture risk however it typically does not consider historical doses. This method has an advantage in that it provides clinicians and patients with information about the extent to which increasing dose is likely to affect the risk of fracture. For example, de Nijs et al. [25] showed that there was an increased fracture risk of about 16% per mg per day. Furthermore, this definition of glucocorticoid exposure assumes that the risk of fracture increases linearly with the increase of dose. This means that it is impossible to examine whether the change to the risk of fracture tapers at some value of daily dose.

The “cumulative dose” method assumes that all current and prior glucocorticoids have equal impact on fracture risk regardless of how recently they were taken. However, calculating cumulative dose can be difficult which may lead to misclassification and imprecision of estimates. The dynamic regeneration and repair of bone suggests cessation of glucocorticoids might be followed recovery in bone health as shown by Van Staa et al. [10]. The effect of historic doses probably, therefore, has less of an impact on fracture risk compared to more recent doses. Furthermore, the “cumulative dose” method is unable to distinguish between long term, low dose treatments and short-term, high dose treatments. This disadvantage can be overcome by using a multi-variable model and nesting cumulative dose within daily dose such as the models used by Van Staa et al. [46] and De Vries et al. [26]. For example, this allowed for differentiation between patients who had taken between 2.5 and 4.9 mg/day with a total cumulative dose of > 1 g and 15–29.9 mg/day with a cumulative dose of > 1 g. In this case the relative risk (95% CI) were 1.41 (1.23, 1.62) and 2.84 (2.45, 3.30) suggesting the higher dose for short periods of time has a greater impact on fracture risk.

The “time variant” method assumes that the duration spent on glucocorticoids affects fracture risk. Within this model, the majority of results found an increased fracture risk with increasing duration of use. However, the results are difficult to compare as they consider the unit of time as years [20], [21], [27], [29], [47], months [40], [44] and days [45].

Beyond the five models described above, and illustrated in Fig. 1, Sugiyama [41], [42] also included the effect of pulse therapy, initial daily dose and “number of glucocorticoid dose increases” as methods for defining glucocorticoid exposure. Van Staa et al. [9] considered the number of prescriptions received in the past 6 months as a measure of glucocorticoid dose with the participants split into 3 categories (0, 1–2, > 2 prescriptions). They found that patients with 2 or more prescriptions increased their risk by 160% whereas patients with no prescriptions increased their risk by only 30% compared to patients without RA.

None of the models described above considered dose, duration and timing simultaneously, despite it being probable that all are important. Furthermore, no study made a comparison of how recency of glucocorticoid exposure affects the risk of fractures. One novel method that does consider dose, duration, and timing is the weighted cumulative dose model [48]. This method has previously been used to investigate the risk of infection with glucocorticoid therapy [49]. This method weights the dose by how recent it is to the occurrence of the adverse event of interest. A cubic spline curve is fitted allowing the data to define the shape of the weighting curve and hence determine which time points provide the largest effect of the glucocorticoids, in combination with the dosage. Whilst many studies consider the dose-dependent risk irrespective of treatment duration, this method allows risk estimates for any given pattern of glucocorticoid use and can thus allow the comparison of the same dose but taken for different durations. For example, Dixon et al. [49] found that 5 mg prednisolone equivalent taken for 3, 6, 12 months or three years conferred an increased risk of serious infection of 11%, 30%, 55 and 100%, respectively, compared to non-use. This shows that historical doses, and the duration of such use, are important. It also allows consideration of the same dose and duration pattern, but taken at different times with respect to the event of interest, thereby allowing the exploration of recovery from risk (for example, six months at 5 mg/day for the last six months, versus six months at 5 mg/day started a year ago and discontinued six months ago).

Considering the limitations of this review, abstracts were screened and data extracted by a single reviewer. Only the two leading databases were included in the search for publications and papers not written in the English language were also excluded from the review. As the key focus of the publication was to identify the differences in methodology, this was felt to be reasonable. Within the publications, it was unclear whether the glucocorticoids were prescribed for RA or another illness. Despite a possible alternative indication within an RA population, any observed relationship would remain valid unless the effect of glucocorticoid therapy is modified significantly by the indication. Furthermore, this review spans the development of anti-TNF therapy for use in inflammatory conditions. Due to the potential direct impact of biologic therapy on fracture risk, a comparison between studies undertaken before and after 2000 would be useful. However, due to the heterogeneity of the study designs included within this review it is difficult to make a direct comparison within any given method between pre and post 2000 since there maybe unmeasured confounding.

## 5 Summary

There are five main methods by which fracture risk has been attributed to glucocorticoid exposure, none of which consider the dose, duration and timing of treatment. This means risk estimates will rarely consider the complex individual patterns of steroid treatment, and will thus not give an accurate fracture risk assessment for an individual patient. There are now opportunities with advanced analytical methods to incorporate all these factors into a single model, allowing the generation of risk estimates for any given pattern of steroid exposure.

## Disclosures

DER and WGD report no conflicts of interest. EMD has received speaker fees from Eli Lilly. CC has received consultancy fees and honoraria from Alliance for Better Bone Health, Amgen, Eli Lilly, GSK, Medtronic, Merck, Novartis, Pfizer, Roche, Servier, Takeda and UCB. TPvS reports grants on an observational research project from GSK and participation in expert meetings with GSK, Sanofi and Roche, all outside the submitted work.

## Figures and Tables

**Table 1:** Results of literature search.

Ref.	Design	Population type	Comparator population	Study methodology							n	Fracture type	OR/RR/HR/P-value
				CU	EU	DD	CD	ML	TV	OT			
Van Everdingen [11]	Double blinded randomised control trial	RA patients given prednisone	RA patients given placebo	✓							40 + 41	Vertebral fracture	Not signif, OR not given
Furuya [12]	Prospective cohort	RA patients using GC	RA patients not using GC	✓							1733	Vertebral, main non vertebral and any non-vertebral	RR: Vt 1.90 (0.61, 5.94), any Non-Vt 1.69 (1.01,2.83)
Coulson [13]	Retrospective cohort	RA patients, GC use	RA patients, non GC use	✓							8419	Any, vertebral, hip and non-vertebral, non-hip fracture	RR: any fracture 1.325 signif, Vt 1.211 not signif no CI given
Hooyman [14]	Retrospective cohort	Females with RA with fracture of interest	Female with RA without fracture of interest	✓							388	Femur, humerus, pelvis, forearm, vertebral	RR: femur fracture 2.15 (0.79, 4.67)
Orstavik [15]	Retrospective cohort	Early RA patients long term GC use	Early RA patients none or short term GC use	✓							249	Vertebral fracture	OR: 4.15 (1.70,10.07)
Verstraeten [16]	Retrospective cohort	Postmenopausal women with RA	Postmenopausal controls	✓							147	Vertebral fracture	P value < 0.01
Cooper [17]	Case control	RA patients admitted to orthopaedic unit	Community controls 1:2 matched	✓							300 + 600	Hip fracture	OR: 2.5 (1.1, 5.5)
Butler [18]	Case control from cohort	Rheumatology patients taking low dose GC therapy	Rheumatology patients not taking GC therapy	✓							142	Any fracture	P-value < 0.05
Arai^a^[19]	Cross sectional	RA patients	Healthy patients admitted for osteoporosis evaluation	✓							117	Vertebral fracture	OR: 3.82 (3.01, 4.85)
Orstavik [20]	Retrospective cohort	Established RA patients	General population	✓	✓				✓		528	Vertebral fracture	P-values CU & EU < 0.001 RR: TV 1.55 (1.03, 2.33)
Kay [21]	Case control	RA patients with fracture	RA patients no fracture	✓	✓	✓			✓		18 + 18	Stress fracture	P-values: CU 0.32, EU 0.02, DD 0.0003, TV 0.06
Furuya [22]	Prospective cohort	RA patients using GC	RA patients not using GC	✓		✓					9720	Hip fracture	HR: CU not signif. DD: men no change, women 1.03 (1.00, 1.06)
Furuya [23]	Prospective cohort	RA patients using GC	RA patients not using GC	✓		✓					1020	Non-vertebral and vertebral fracture	HR: CU not signif. DD: Vt 1.28 (1.14, 1.45) Non-Vt 1.01 (0.86, 1.18)
Ghazi [24]	Case control	RA patients	General population	✓		✓					101 + 303	Vertebral fracture	OR: CU 0.17 (0.04, 0.66) DD not reported
de Nijs [25]	Cross sectional	RA patients using oral GC on daily basis ≥ 1 month	RA patients not using GC matched 1:1	✓		✓	✓				410	Vertebral fracture	OR: CD 1.00 no CI CU 4.31 (1.13, 16.47)⁎ DD: 1.16 (1.05, 1.28)⁎
De Vries [26]	Retrospective cohort	RA patients with current GC use	RA patients with past GC use	✓		✓	✓	✓			191,752	Osteoporotic, hip and vertebral fracture	RR: Osteo CU 1.68 (1.6, 1.76) DD < 7.5 mg 1.60 (1.50, 1.71) 7.5–15 mg 2.15 (1.97, 2.34) CD < 1g^b^ 1.38 (0.59, 3.22) 1–5g^b^ 8.12 (5.19, 12.74)
Nampei [27]	Prospective cohort	RA patients taking GC	Different strength or duration of GC use	✓		✓			✓		209	Any, vertebral and “lower leg and pelvis” fracture	OR: all DD 1.174 (1.054, 1.306) TV 1.095 (1.015, 1.181) pelvis DD 1.195 (1.043, 1.370) TV 1.131 (1.033, 1.239)
Maghraoui [28]	Cross sectional	RA patients using GC	RA patients, non-current use and different category of cumulative use	✓			✓				172	Vertebral fracture	P-value CD < 0.0001
Mazzantini [29]	Retrospective cohort	GC users ≥ 6 months	Never use of GC	✓					✓		2359	Osteoporotic fracture	P-value CU < 0.02 TV > 5 years < 0.001 < 2 and 2–5 years > 0.05
Michel [30]	Retrospective cohort	RA patients with GC use	RA patients not using GC		✓						395	Any fracture	RR: 1.9, P-value 0.026
Wright [31]	Prospective cohort	Arthritis patients using GC	Patients not using GC		✓						147,657	Any, hip and clinical spine fracture	P-value > 0.05
Arai^a^[19]	Prospective cohort	RA patients using GC	RA patients not using GC		✓						112	Vertebral fracture	P-value < 0.05 for age groups 50–54 and 60–64
Saville [32]	Cross sectional	RA patients taking GC	RA patients not taking GC		✓						164	Vertebral fractures	P-value > 0.05
Laan [33]	Cross sectional	RA patients taking GC	RA patients not taking GC		✓						77	Vertebral fractures	P-value 0.03
Vis [34]	Prospective cohort	Established RA patients using GC	RA patients not on GC		✓						102	Vertebral and non-vertebral fracture	P-value: V 0.04, Non-V 0.30
Lapi [35]	Retrospective cohort	Those taking GC	Those not taking GC		✓						271,121	Osteoporotic and hip fracture	OR: Osteo: male 1.39 (0.98, 1.97) female 1.69 (1.42, 2.01)
Peel [36]	Case control	Postmenopausal women with RA	Population based		✓						76 + 347	Vertebral fracture	Non signif
Ochi [37]	Prospective cohort	RA patients with fracture	RA patients no fracture		✓	✓					9987	Distal radius	HR: DD 1.07 (1.01, 1.13) EU: higher % GC users in fracture group
Saag [38]	Case control	Early RA, > 1 year CS use	Early RA, 1:1 matched, no GC use		✓	✓					112 + 112	Any fracture	P-value EU > 0.05 5–10 mg/d 4.5 (2.1, 9.6)
Lems [39]	Case control	RA patients treated with GC on daily basis ≥ 1 month	Rheumatology patients not using GC		✓	✓					52 + 55	Vertebral or peripheral fracture	P-values: Vt 0.03, peripheral > 0.05
Baskan [40]	Cross sectional	RA patients	Healthy patients		✓	✓			✓		156	Vertebral fracture	P-value > 0.05
Sugiyama [41]	Prospective cohort	Rheumatic disease patients on high dose GC	Rheumatic disease patients not on GC		✓	✓	✓			✓	2631	Vertebral fracture	HR: EU 8.16 (1.09, 60.86) DD 2.03 (1.43, 2.88) CD 0.88 (0.84, 0.93) OT various
Sugiyama [42]	Prospective cohort	Early RA patients high dose GC	Early RA patients not on GC			✓	✓			✓	700	Vertebral fracture (symptomatic)	HR: DD 1.24 (1.16, 1.33) CD 0.92 (0.91, 0.94) OT various
Sinigaglia [43]	Retrospective cohort	Established RA patients GC use	Established RA patients no GC use				✓				925	Vertebral fracture	OR: CD 1.03 (1.006, 1.07)
Lespessailles [44]	Cross sectional	RA patients using GC	Non GC users				✓		✓		146	Vertebral and any fracture	OR: any: CD 0–5 vs 15 + 4.04 (1.5, 11.2) TV 0–12 vs 60 + months 2.8 (1.07, 7.3)
Angeli [45]	Cross sectional	RA patients with high cumulative dose	RA patients with low cumulative dose				✓		✓		551	Vertebral fracture	P-values all > 0.05
Van Staa [46]	Retrospective cohort	Oral GC patients	No GC prescription in past 3 months					✓			191,752	Osteoporotic, hip and vertebral fracture	RR: Vt: various results: e.g. DD < 2.5, CD < 1 2.11 (0.87, 5.10) DD < 2.5, CD > 1 3.22 (2.09, 4.95)
Michel [47]	Case control	RA patients with fracture	RA patients without fracture						✓		226 + 884	Any fracture	OR: 1.07 (1.04, 1.11)
Van Staa [9]	Retrospective cohort	RA patients	Population based 1:3 matched							✓	121,045	Osteoporotic, hip and vertebral fracture	RR: N. prescriptions 0 prescript 1.5 (1.2, 1.9) 1–2 prescript 2.9 (1.9, 4.4) >2 prescript 5.5 (4.4, 6.8)

Abbreviations: CU = current use, EU = ever use, DD = daily dose, CD = cumulative dose, ML = multi-level, TV = time variant, OT = other, OR = odds ratio, RR = relative risks, HR = hazard ratio, RA = rheumatoid arthritis, GC = glucocorticoid(s), Vt = vertebral fracture, Non-Vt = non-vertebral fracture, Osteo = osteoporotic fracture, signif = significant, N. = number.

a The two results by Arai [19] were produced in the same paper in a study which originally started as a cross sectional study but extended in most patients to a Prospective Cohort study.

⁎ Crude odds ratios.

## Appendix 1: Search terms used

The explode option (exp) was used for terms of particular interest where the papers of interest may fit into multiple subcategories.

The terms categorised under glucocorticoids included:

• Triamcinolone
• Dexamethasone
• Prednisone
• Prednisolone
• Methylprednisolone
• Budesonide
• Bethamethasone
• Cortisone
• Glucocorticoids
• exp Corticosteroids
• Terms for the drug adjacent within 2 words (adj2) to methods of taking the drug, where terms for the drug included glucocorticoid*, Glucocorticosteroid*, Corticosteroid* and steroid* and methods of taking the drug included therap*, prescript*, use*, treat*, oral, and tablet.
• The terms categorised under fracture included:
• Fracture*
• Terms categorised under rheumatoid arthritis included:
• Rheumatoid arthritis
• exp. Arthritis, Rheumatoid
• Inflammatory polyarthritis
• Inflammatory arthritis
